# The Composites of PCL and Tetranuclear Titanium(IV)–Oxo Complex with Acetylsalicylate Ligands—Assessment of Their Biocompatibility and Antimicrobial Activity with the Correlation to EPR Spectroscopy

**DOI:** 10.3390/ma16010297

**Published:** 2022-12-28

**Authors:** Julia Śmigiel, Piotr Piszczek, Grzegorz Wrzeszcz, Tomasz Jędrzejewski, Patrycja Golińska, Aleksandra Radtke

**Affiliations:** 1 Faculty of Chemistry, Nicolaus Copernicus University in Toruń, Gagarina 7, 87-100 Toruń, Poland; 2 Faculty of Biological and Veterinary Sciences, Nicolaus Copernicus University in Toruń, Lwowska 1, 87-100 Toruń, Poland

**Keywords:** titanium(IV) complex, oxo-complex, composite, antimicrobial activity, cytotoxicity, poly(ε-caprolactone), EPR analysis

## Abstract

In our research, we have focused on the biological studies on composite materials produced by the dispersion of titanium(IV)–oxo complex (TOC) with acetylsalicylate ligands in a poly(ε-caprolactone) (PCL) matrix, which is a biodegradable thermoplastic polymer increasingly used in the production of medical devices. Using PCL as a matrix for the biologically active compounds, such as antimicrobial agents, antibiotics or other active medical substances, from which these individuals can be gradually released is fully understable. Composites of PCL + nTOC (n = 10, 15 and 20 wt.%) have been produced and, in such a form, the biological properties of TOCs have been estimated. Direct and indirect cytotoxicity studies have been performed in vitro on L929 and human umbilical vein endothelial cells (HUVEC) cell lines. The antibacterial and antifungal activity of the PCL + TOC samples have been assessed against two *Staphylococcus aureus* (ATCC 6538 and ATCC 25923) reference strains, two *Escherichia coli* (ATCC 8739 and ATCC 25922) reference strains and yeast of *Candida albicans* ATCC 10231. Obtained results have been correlated with electron paramagnetic resonance (EPR) spectroscopy data. We could conclude that photoexcitation by visible light of the surface of PCL + nTOC composite foils lead to the formation of different paramagnetic species, mainly O^−^, which slowly disappears over time; however, their destructive effect on bacteria and cells has been proven.

## 1. Introduction

Due to the large number of bacterial strains resistant to many of the currently used drugs and disinfectants, our previous research was focused on the synthesis and evaluation of the microbiocidal activity of tri and tetranuclear oxo–titanium(IV) complexes (TOCs). Their biological properties have been checked in the form of composite foils prepared by the dispersion of TOCs in poly(methyl methacrylate) (PMMA) matrix. The results of that research suggested that composites produced, due to their bactericidal activity, can be used in the elimination of microbiological contamination in various areas of our life [1,2]. We have also carried out the synthesis and structural characterization of titanium(IV) oxo-complex with an acetylsalicylate ligand, with the overall formula [Ti_4_O_2_ (O^i^Bu)_10_(asp)_2_]·H_2_O (**1**), where asp is the aspirine ligand or acetylsalicylate ligand [3]. The structure of (**1**) was solved by the single crystal X-ray diffraction method and confirmed by vibrational (IR and Raman) spectroscopy and 13C NMR solid state spectroscopy. The analysis of the UV-Vis DRS spectrum proved that the maximum absorption position is about 395 nm, thus in the visible range, and the value of the HOMO-LUMO energy gap is 2.35 eV. We have performed tests of the photocatalytic activity of composite films produced by the dispersion of (**1**) microspheres as inorganic blocks in a polycaprolactone (PCL) matrix (PCL + (**1**)). The experiments, based on visible light photoinduced decolorization of the methylene blue solution, showed good and stable photocatalytic activity of the PCL + (**1**) composite films. This activity does not change during the PCL matrix degradation process, which may result from the structural stability of the oxo cluster of (**1**) [3]. Having a comprehensive chemical knowledge of the new oxo-complex, [Ti_4_O_2_ (O^i^Bu)_10_(asp)_2_]·H_2_O, we have decided to investigate its biological properties, especially since the ligands in this Ti(IV) complex are known for their antithrombogenic properties. However, before starting to study the detailed biological properties of biomaterials, it is important to assess their biocompatibility, or at least exclude cytotoxicity. It is also worth examining the microbicidal potential of the biomaterial—it should be remembered that contact of the biomaterial with the recipient’s organism often leads to the formation of a bacterial biofilm. Therefore, it is good to know whether the biomaterial enhances or blocks the formation of bacterial biofilm and, consequently, the formation of inflammation.

In the present study, in vitro biocompatibility of PCL and PCL + (**1**) composite samples were evaluated using two cell lines, L929 fibroblasts and human umbilical vein endothelial cells (HUVEC cells), which represent cells essential for the proper functioning of materials with antithrombotic properties. L929 cells are a commonly-used model for in vitro cytotoxicity studies [4]. Moreover, fibroblasts are the main cells in loose connective tissue, which are commonly found in the peri-implant soft tissue and are essential to prevent epithelial ingrowth [5]. HUVEC cells are one type of endothelial cells that are very often used in research associated with the function of blood vessel endothelium. The endothelium, which resides at the interface between the blood and surrounding tissues, plays an integral role in the haemostatic system. Depending on specific tissue needs and local stresses, endothelial cells are capable of evoking either antithrombotic or prothrombotic events [6]. Moreover, endothelial cells play an important role in immune response, coagulation, growth regulation, modulation of blood flow and production of the extracellular matrix. After the material is deposited into the blood vessel, its surface is in direct contact with the endothelial layer. The re-endothelialisation onto the inner layer of the material is also a very important step for the vascular reprogram [7]. The interaction between the antithrombotic material and endothelial cells, therefore, is of great importance for the effectiveness of the treatment. Considering the research on microbiocidal activity, it should be noted that, despite our noticeable influence on conducting research in this area, the antimicrobial activity of multinuclear oxo–titanium(IV) complexes has not been fully explored so far [1,2]. Not only our group, but also Svensson et al., revealed the antibacterial activity of the oxo-complexes that consisted of {Ti_4_O_2_} cores stabilized by two triclosan ligands against *S. aureus* [8].

Taking into account all the above, we set ourselves the goal of checking the basic biological properties of composite systems formed by the dispersion of a new titanium(IV) complex compound, which we recently obtained and described, in a PCL matrix. Moreover, we wanted to link biological activity with the potential formation of active oxygen species on the surface of composites during irradiation with visible light. In the future, studied composite systems containing Ti(IV) oxo-acetylsalicylates may be used as coatings for cardiac stents, whose task will be the gradual release of antithrombogenic substances.

## 2. Materials and Methods

### 2.1. Materials 

Titanium(IV) isobutoxide (Aldrich, St. Louis, MO, USA) and acetylsalicylic acid (ASA, Aldrich, St. Louis, MO, USA) were used without further purification, as purchased commercially. All solvents used in the synthesis, i.e., tetrahydrofuran (THF) and isobutanol (HO^i^Bu), were distilled before their use and stored in argon atmosphere. The processes of Ti(IV)–oxo complex synthesis were carried out using the standard Schlenk technique, in the inert gas atmosphere (Ar) and at room temperature (RT).

### 2.2. Synthesis of the Tetranuclear Ti(IV)–Oxo Complex (1) Stabilized by Acetylsalicylate Ligands

The synthesis of the tetranuclear Ti(IV)–oxo complex stabilized by acetylsalicylate ligands (1) has been carried out according to our procedure reported earlier [3]: 0.88 mmol of acetylsalicylic acid was added to the solution of 3.52 mmol titanium(IV) isobutoxide in 2 mL of THF/HO^i^Bu (1:1) in order to obtain a clear yellow solution. The solution was left for crystallization, and, after three days, light yellow crystals of [Ti_4_(μ_4_-O)(μ-O)(asp)_2_(BuiO)_10_]·H_2_O were isolated from the mother liquor, with the yield 74.00%.

### 2.3. Preparation of the Composite Foils 

Foils of composites based on polycaprolactone (PCL), i.e., PCL + TOC, have been prepared in the same way, as it was presented in our earlier works [1,2,3,9]. The TOC powder portion (acting as 10,15 or 20 wt.% of the reaction mixture) was dispersed (with the use of an ultrasonic bath) in the solution, which was obtained by dissolving 1 g PCL in 5 cm^3^ of THF. The composite films were produced by slow solvent evaporation in a glove box at room temperature

### 2.4. Biocompatibility Study of PCL and PCL + (1) Composite Materials

#### 2.4.1. Samples Sterilization and Preparation for Cell Culture

The specimens of PCL and PCL + (1) measuring 6 mm × 6 mm (for the direct cytotoxicity assay) or 25 mm × 25 mm (for the extract cytotoxicity method) were sterilized using an immersion in 70% (*v*/*v*) ethanol/water solution for 20 min followed by UV irradiation for 30 min for each side of the foils. Then, the samples were washed three times with PBS to remove the residual ethanol.

#### 2.4.2. Cell Culture

For these studies, murine fibroblast cell line L929 and Human Umbilical Vein Endothelial Cells (HUVEC) were used. The L929 cell line was purchased from American Type Culture Collection (Manassas, VA, USA) and cultured in RPMI 1640 medium supplemented with 10% heat-inactivated fetus bovine serum (FBS) and 1% mixture of antibiotics (streptomycin and penicillin; all compounds from Merck KGaA, Darmstadt, Germany). HUVEC cells were obtained from Thermo Fisher Scientific (Waltham, MA, USA) and cultivated in Medium 200 supplemented with Low Serum Growth Supplement (LSGS) and antibiotics. HUVEC cells were cultivated in the culture flasks coated with Attachment Factor Protein containing gelatin, which promotes attachment and growth of microvascular endothelial cells (all compounds used for the culture of HUVEC cells were purchased from Thermo Fisher Scientific). Both cell lines were maintained at 37 °C in a humidified atmosphere with 5% CO_2_. The cells were trypsinized using 0.25% trypsin-EDTA solution (Merck KGaA).

#### 2.4.3. Cytotoxicity Assays

A potential cytotoxicity of PCL and PCL + (1) foils was evaluated according to the ISO 10993-5 and ISO 10993-12 standards using two different methods: extract test and direct contact to the materials. 

##### Extract Assay

For extract tests, after sterilization, the samples were placed into 6-well culture plates. The ratio of the PCL and PCL+ (1) films to extraction vehicle was 3 cm^2^/mL. For sample extraction, the plates were maintained in an incubator at 37 °C and 5% CO_2_ for 72 h in the complete culture medium (RPMI 1640 for L929 cells or Medium 200 for HUVEC cells). The supernatant fluids were withdrawn and centrifuged (2000× *g* for 15 min) to prepare the extraction medium, then sterilized using 0.22 µm filter and refrigerated at 4 °C before the cytotoxicity tests were conducted. The culture media without foils were incubated in the same conditions and used as non-cytotoxic controls for cell treatment. For the evaluation of extract cytotoxicity, 5 × 10^3^ cells were seeded in 96-well tissue culture plates (in the case of HUVEC cells, the wells were pre-coated with Attachment Factor Protein before seeding) and they were pre-incubated for 24 h. Thereafter, the extracts were diluted with appropriate culture medium to five testing concentrations, namely 10% extracts (dilution factor 1:10), 16.7% extracts (dilution factor 1:6), 33.3% extracts (dilution factor 1:3), 50% (dilution factor 1:2) and 100% extracts, according to the recommendation in Kubásek et al. [10]. The cells were stimulated with the extracts for 24, 48 and 72 h. The cell viability was evaluated using MTT (3-(4,5-dimethylthiazolyl)-2,5-diphenyl-tetrazolium bromide; Merck KGaA, Darmstadt, Germany) assays. After cell treatment, 100 µL/well of MTT solution at a final concentration of 0.5 mg/mL was added to each well, the culture plates were incubated at 37 °C for 3 h followed by the addition of 100% dimethyl sulfoxide (DMSO; 100 µL/well). Absorbance values were measured at 570 nm and referenced at 630 nm using a Synergy HT Microplate Reader (BioTek Instruments, Winooski, VT, USA). The results were shown as a percentage of cells cultivated in the corresponding concentrations of culture control media that were treated in the same way as extracts (served as 100%). 

##### Direct Cytotoxicity

For the evaluation of the direct cytotoxicity, the sterilized PCL and PCL + (1) samples were placed in individual wells of a 24-well plate. Then, 25 µL of a cell suspension at 1 × 10^3^ cells/μL was seeded on the samples and incubated for 4 h at 37 °C and 5% CO_2_ to allow cells to attach to the surface of materials. Thereafter, the wells were flooded with 1 mL of an appropriate complete culture medium and incubated at 37 °C and 5% CO_2_ for 24, 48 and 72 h. Then, the composite foils were washed with PBS and 500 µL of the MTT solution (0.5 mg/mL) was added to each well. After 3 h of incubation at 37 °C, 500 µL of DMSO was added. The absorbance was measured at 570 nm and referenced at 630 nm using a microplate reader. 

#### 2.4.4. SEM Analysis 

For the imaging study and analysis of cell morphology, the cells were incubated on the surface of PCL + (1) 10 wt.% foils for 24, 48 and 72 h. Thereafter, scanning electron microscopy (SEM; Quanta 3D FEG; Carl Zeiss, Göttingen, Germany) analyses were conducted. After the selected incubation time, the specimens were washed with PBS, fixed in a 2.5% *v/v* glutaraldehyde and dehydrated in a graded series of ethanol (50%, 75%, 90% and 100%).

#### 2.4.5. Statistical Analysis in the MTT Assay

The GraphPad Prism 7.0 software (GraphPad Software Inc., San Diego, CA, USA) was used for statistical analyses. All values are reported as means ± standard error (SEM) and were analyzed using analysis of variance (ANOVA) followed by Tukey’s multiple comparisons test, with the level of significance set at *p* < 0.05.

### 2.5. Antibacterial and Anticandidal Activity of PCL + (1) Composite Materials 

Antimicrobial activity of PCL and PCL + TOC composites (30 × 30 mm) was studied against Gram-positive (*Staphylococcus aureus* ATCC 6538 and *S. aureus* ATCC 25923) and Gram-negative (*Escherichia coli* ATCC 8739 and *E. coli* ATCC 25922) bacterial strains, and yeasts (*Candida albicans* ATCC 10231) that were grown for 24 h at 37 °C in Triptic Soy Broth (TSB, Becton Dickinson, Franklin Lakes, NJ, USA) for bacteria and Sabouraud Dextrose Broth (SDB, Becton Dickinson) for yeasts. All the reference strains were obtained from the American Type Culture Collection (Manassas, VA 20110, USA). The composites were surface sterilized for 15 min (each side) using a UVC lamp for 15 min in the laminar hood (Bioquell, Hampshire, UK). Polymer samples were then exposed to visible light (indoor light), placed in wells of sterile 6-well plates (Nest) and poured with 2 mL microbial inoculum (1.0–3.6 × 10^6^ colony forming units (c.f.u) cm^3^/mL^−1^) in sterile deionized water. The density of microbial inoculum (0.5 McFarland scale) was established using a densitometer (Biosan, Latvia) and diluted 100-times to obtain final concentrations. Plates were incubated for 24 h at 37 °C in a humid atmosphere and shaken (100 rpm) conditions. Samples were then mixed well by pipetting, and suspensions were collected in sterile tubes and vortexed. Subsequently, serial ten-fold dilutions of microbial suspensions in sterile distilled water were prepared and each dilution (100 μL) was aseptically spread over the surface of Triptic Soy Agar (TSA, Becton Dickinson, NJ, USA) and Sabouraud Dextrose Agar (SDA, Becton Dickinson) in Petri plates. Inoculated plates were incubated for 24 h at 37 °C and examined for c.f.u. The positive control was the microbial inoculum in the well without specimen. Assays were performed in triplicate. The antimicrobial activity was determined based on the reduction (R) factor calculated according to the formula:

𝑅 = Ut **−** At (1)

where Ut is the common logarithm of the number of microorganisms recovered from the untreated microbial suspension after 24 h, At is the common logarithm of the number of microorganisms recovered from the microbial inoculum treated with PCL or PCL + TOC after 24 h. Reduction factor (R) ≥ 2 indicates biocidal activity of a specimen (at least 99% reduction of microbial growth).

### 2.6. Electron Paramagnetic Resonance Research

The electron paramagnetic resonance (EPR) spectroscopy has been used in order to confirm paramagnetic radicals (reactive oxygen species) formation on the surface of the investigated samples. Measurements were carried out using an X band EPR SE/X-2541M spectrometer (Radiopan, Poznań, Poland) with a 100 kHz modulation. The microwave frequency was monitored with a frequency meter. The magnetic field was measured with an automatic NMR-type JTM-147 magnetometer (Radiopan, Poznań, Poland). Measurement conditions: microwave frequency: ca. 9.33 GHz; microwave power: 2–58 mW; modulation amplitude: 0.25–1 mT; sweep: 10–50 mT; sweep time: 4 min.; time constant: 0.1 s; receiver gain: 2–5 × 10^5^. The measurements were performed for cut films of PCL + TOC composites at room temperature for fresh samples and after some time, up to five months.

## 3. Results

Synthesized Ti(IV) oxo-complex (1), obtained on the basis of the procedure described in our report [3], has been checked in terms of its structure and purity using spectroscopic methods. This compound has been used to prepare foils of composites based on polycaprolactone (PCL), i.e., PCL + (1), as it was presented in our earlier work [9]. Such prepared foils have been applied in all analyses.

### 3.1. Cytotoxicity Evaluation of PCL/PCL + (1)

**Figure 1:**
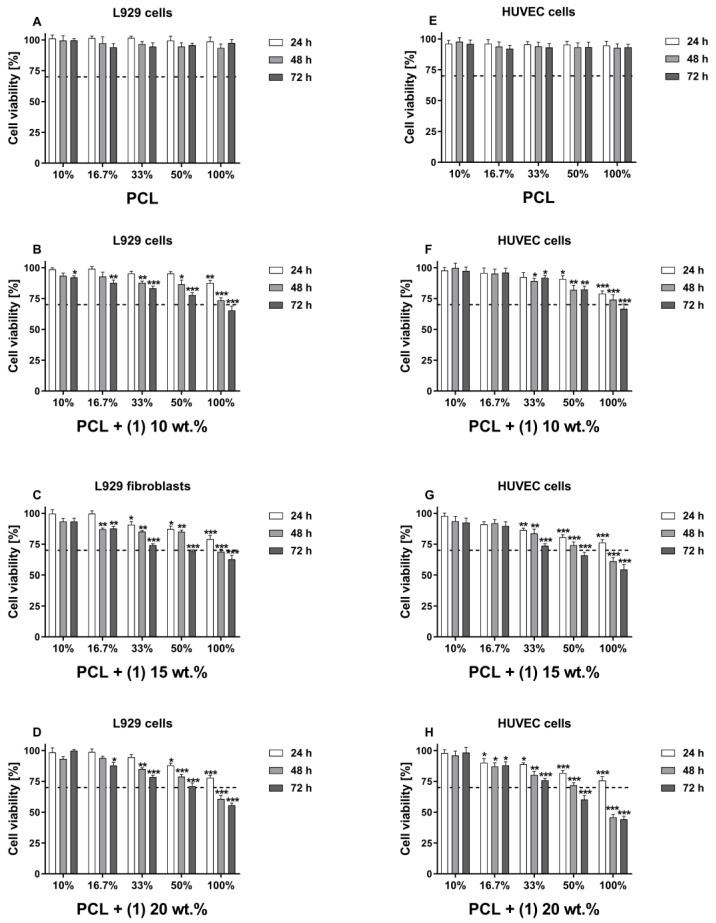
Cytotoxicity of the extracts from PCL and PCL + (**1**) samples containing the different concentrations of TOC that were estimated for L929 fibroblasts (**A**–**D**) and human umbilical vein endothelial (HUVEC) cells (**E**–**H**). Cells were incubated in the different concentrations of extracts (10%, 16.7%, 33%, 50% and 100%) for 24, 48 and 72 h. Cell viability (estimated using MTT assay) was presented as percentage ± SEM. of the control cells cultivated in corresponding concentrations of culture control media that were treated in the same way as extracts. Asterisks show significant differences between the cells cultured in control media (served as 100%) and the cells incubated with the extracts (*** *p* < 0.001; ** *p* < 0.01; * *p* < 0.05). Dash lines present potential cytotoxicity of the extraction media when the cell viability decreases below 70% according to ISO norms.

The potential cytotoxicity of the tested foils of PCL and PCL + (**1**) was evaluated using two different methods: extract test and direct contact to the samples. The extract assay estimated the cytotoxicity of any leachable products from the material after 72 h-lasting of incubation in culture media. As it can be seen in Figure 1A,E, the incubation of both L929 and HUVEC cells in all tested concentrations of extracts derived from PCL specimens did not decrease the cell viability in all time variants. In contrast, the cell viability was significantly reduced following the PCL-/PCL + (**1**)-derived extraction media challenge in time-dependent and concentration-dependent manners. According to the ISO standards, the material is considered as cytotoxic if it decreases cell viability below 70%. Among the tested extracts, the statistically significant cytotoxic effect on L929 fibroblasts was only observed for the cells incubated with the 100% extract derived from PCL + (**1**) 15 wt.% samples during 72 h of incubation (Figure 1C; 62.8 ± 3.2%) and from PCL + (**1**) 20 wt.% specimens after 48 h and 72 h of stimulation (Figure 1D; 60.8 ± 2.9% and 55.7 ± 1.7%, respectively). In the case of HUVEC cells, their viability was reduced below 70% when the cells were incubated with 100% PCL + (**1**) 15 wt.% TOC-derived extract for 48 and 72 h (Figure 1G; 61.1 ± 3.0% and 54.5 ± 4.0%, respectively) and for the cells treated with the 100% extract from PCL + (**1**) 20 wt.% foil for 48 and 72 h (Figure 1H; 45.9 ± 2.2% and 44.3 ± 2.4%, respectively). The statistically significant cytotoxic effect was also observed for HUVEC cells incubated with 50% extract from PCL + (**1**) 20 wt.% for 72 h (Figure 1H; 60.3 ± 3.3%).

**Figure 2:**
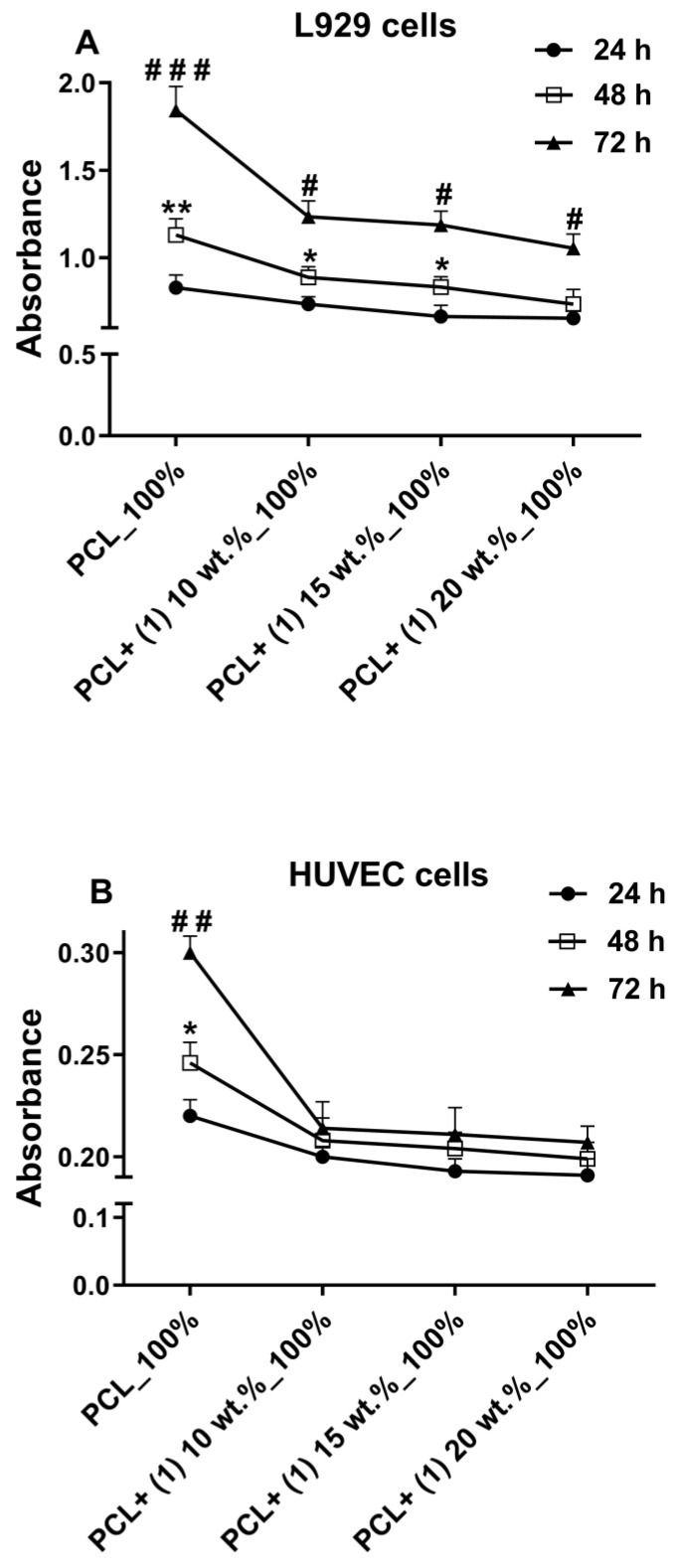
Absorbance values ± SEM measured for L929 fibroblasts (**A**) and HUVEC cells (**B**) incubated with undiluted extracts derived from PCL and PCL + (**1**) samples that reflect the proliferation rate of viable cells over time (measured using an MTT assay). Cells were incubated in 100% extracts for 24, 48 and 72 h. Asterisks show statistical significances between cells treated with extract for 24 and 48 h (* *p* <0.05; ** *p* < 0.01), whereas hash marks present differences between 48 and 72 h (# *p* < 0.05; ## *p* < 0.01; ### *p* < 0.001).

The results presented in Figure 1 show a cytotoxic effect of the undiluted extracts derived from PCL/ PCL + (**1**) foils both on the L929 and HUVEC cells, especially after 72 h of incubation. This effect was compared with the viability of cells cultured in the control medium that was served as 100%. However, slightly different conclusions followed from this phenomenon when the results of viability of cells incubated with 100% extracts were demonstrated as the changes in absorbance values measured over time. As shown in Figure 2A, L929 cells incubated with PCL-/PCL + (**1**)-derived undiluted extract, such as the cells treated with the PCL-derived extract, proliferated over time, in what was observed as an increase in absorbance level. However, this effect was weaker than for PCL samples. Nevertheless, despite the cytotoxic activities of undiluted extracts from PCL/PCL + (**1**) specimens presented in Figure 1, an increase in the L929 cell proliferation level was observed between 48 h and 72 h for all PCL/PCL + (**1**) samples containing different concentration of acetylsalicylic acid (*p* < 0.05). This effect was also noticed between 24 h and 48 h, except for PCL + (**1**) 20 wt.% samples. In contrast, the increase in proliferation level of HUVEC cells over time was observed only for the PCL-derived undiluted extracts (Figure 2B).

**Figure 3:**
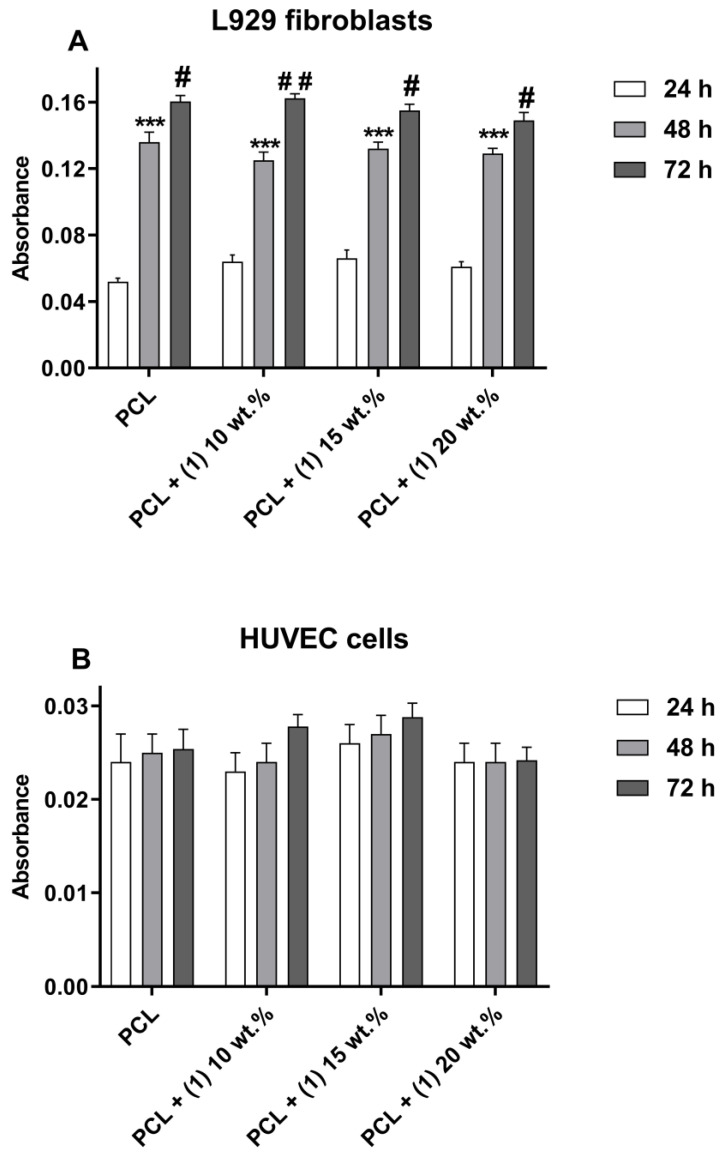
The viability of L929 fibroblasts (**A**) and HUVEC cells (**B**) directly cultured on the surfaces of PCL and PCL + (**1**) for 24, 48 and 72 h. The absorbance values are expressed as means ± SEM of five independent experiments. Asterisks indicate significant differences in the cell viability between 24 and 48 h (*** *p* < 0.001), whereas hash marks between 48 and 72 h (# *p* < 0.005; ## *p* < 0.010).

Figure 3 presents the direct physical effect of the tested foils’ surfaces on the viability of L929 fibroblasts and HUVEC cells. It is worth noticing that, with an increase of the incubation time, more L929 fibroblasts proliferated on all the tested materials (Figure 3A). Analysis of this data revealed that the PCL/PCL + (**1**) samples induced similar levels of L929 cell proliferation on the foils’ surfaces in comparison with PCL specimens. In contrast, all tested materials did not induce the proliferation of HUVEC cells, since the number of these cells growing on the foils’ surfaces did not change over time (Figure 3B).

**Figure 4:**
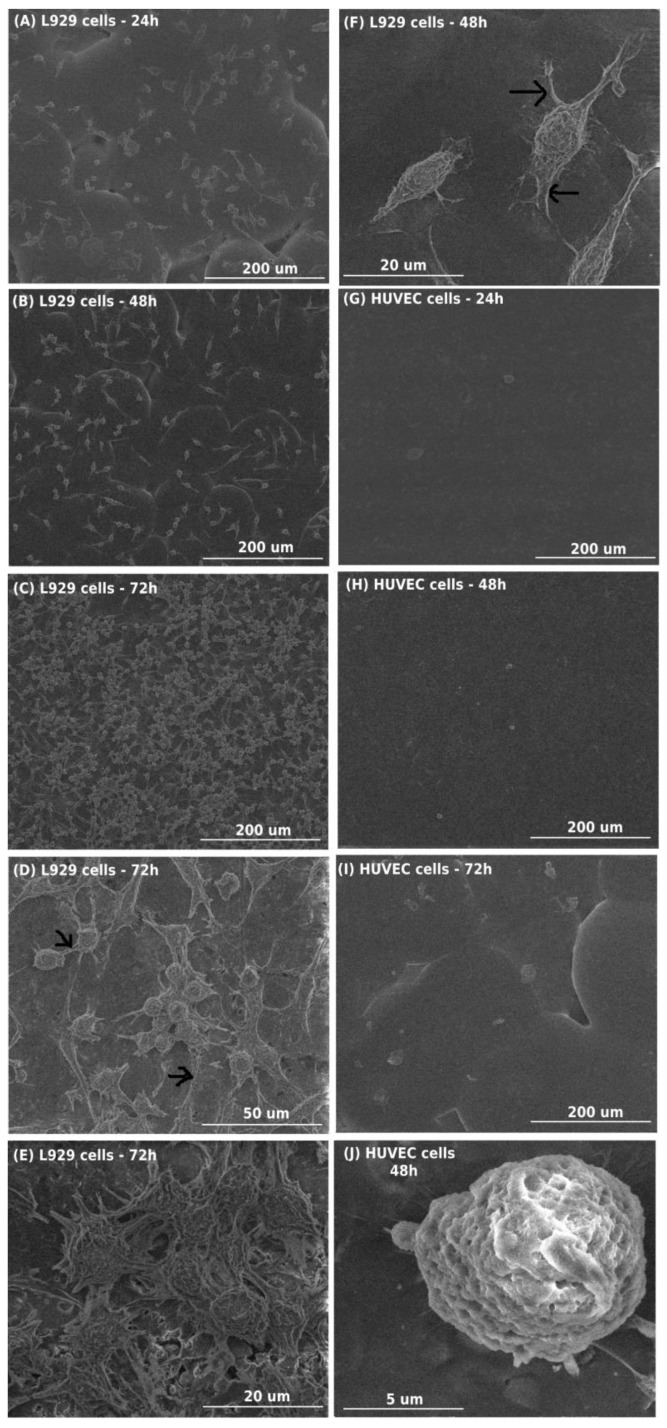
Scanning electron microscopy micrographs showing L929 fibroblasts (**A**–**C**) and HUVEC cells (**G**–**I**) cultured on the surfaces of the PCL + (**1**) 10 wt.% for 24, 48 and 72 h. Cell line names, time of incubation and image scales are presented in the figures. Arrows indicate filopodia spread between the cells (**D**) or filopodia attaching cells to the foil’s surface (**F**). Figure (**E**) presents L929 cells producing the extracellular matrix and growing on top of each other. Figure (**J**) shows rounded shape of HUVEC cells.

Comparative SEM images of L929 fibroblasts and HUVEC cells cultured on the surface of PCL + (**1**) 10 wt.% are shown in Figure 4. Analysis of these micrographs confirmed the results from the MTT assay and clearly indicated that L929 cells cultured on the presented specimens effectively attached to the surfaces and several of the growing cells increased over time (Figure 4A–C). These results also demonstrated that L929 fibroblasts cultured on the PCL + (**1**) 10 wt.% had an elongated shape (Figure 4F), produced the extracellular matrix and grew on top of each other after 72 h (Figure 4D–E). Moreover, L929 fibroblasts created numerous filopodia, formed between cells (Figure 4D), that also attached cells to surfaces (Figure 4B,F). In the case of HUVEC cells, the number of cells that effectively attached to the surface of PCL + (**1**) 10 wt.% was very low, and the number of growing cells did not change over time (Figure 4G–I). Moreover, HUVEC cells had rounded shapes (Figure 4J).

### 3.2. Antimicrobial Activity of PCL + (1) Composite Materials

The results of antimicrobial activity of PCL and PCL + (**1**) are shown in Table 1. Generally, this assay revealed that PCL polymer enriched with TOC grains decreased the number of tested microorganisms compared to polymer (PCL), and that Gram-positive bacteria were more sensitive to PCL + (**1**) at all tested concentrations of grains. The biocidal effect (R ≥ 2) of PCL + (**1**) composites was observed against both *S. aureus* strains (R in the range of 4.5–4.6), but not against *E. coli* strains and *C. albicans*. The enrichment of PCL with TOC grains (15 and 20 wt.% TOC) resulted in biocidal activity of composites against *E. coli* strains (reduction index in the range of 2.1–2.8 and 2.0–3.7, respectively). In the case of *C. albicans,* higher TOC contents cause a static effect of growth, but not a biocidal one (Table 1). 

### 3.3. EPR Studies

**Figure 5:**
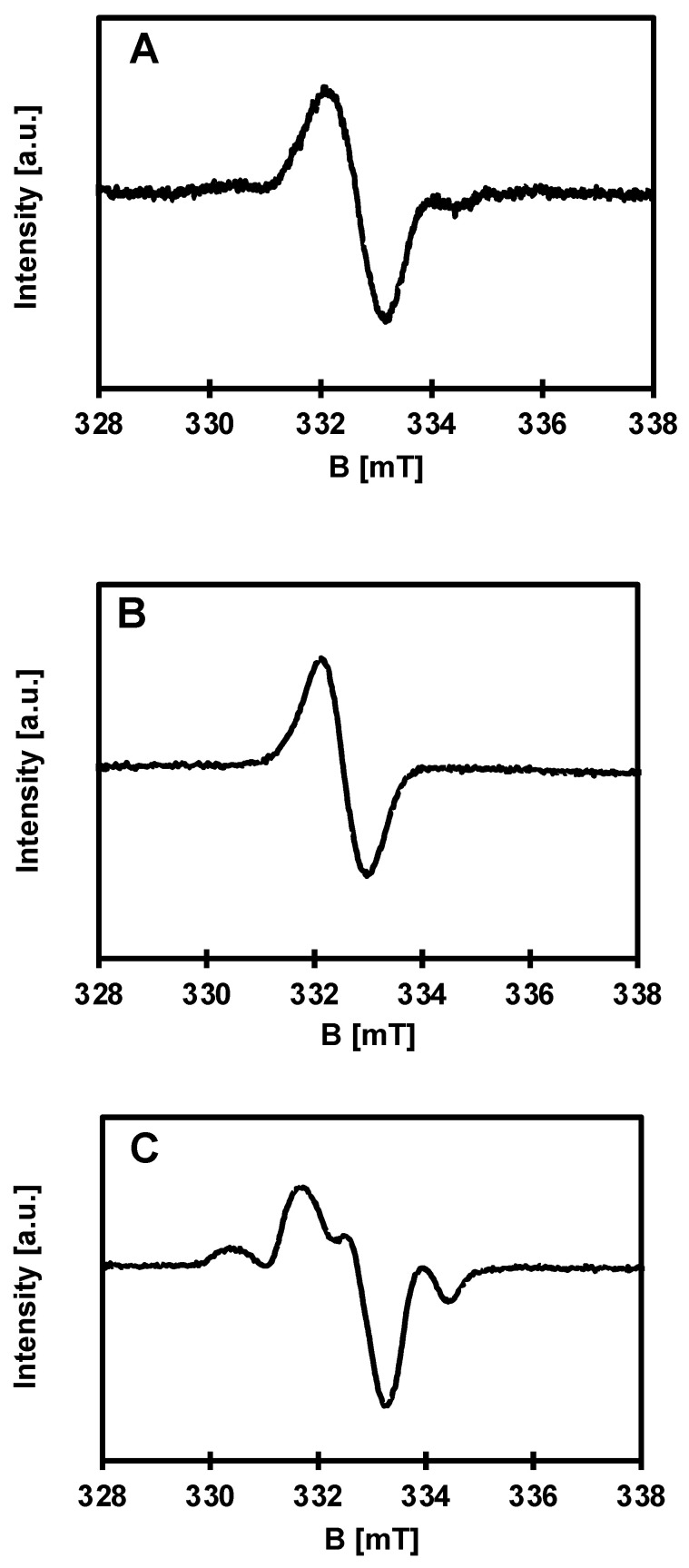
EPR spectra of PCL with 10 (**A**), 15 (**B**) and 20% (**C**) of (**1**) samples. Some experimental conditions: cut foil, room temperature, microwave frequency: 9.32711 (**A**), 9.32518 (**B**), 9.32473 (**C**) GHz; microwave power: 40 mW; modulation amplitude: 1 mT; sweep: 20 mT; sweep time: 4 min.; time constant: 0.1 s; receiver gain: 4 × 10^5^ (**A**,**B**), 2 × 10^5^ (**C**). Measurement time: fresh sample (**A**), after a month (**B**) and fresh sample (**C**).

EPR spectroscopy was used to detect paramagnetic species on the surface of the synthesized materials. Pure PCL polymer shows no EPR signal. However, paramagnetic centers are found in all composite samples of PCL + 10, 15 and 20 wt.% of (**1**), as well as in pure TOC (**1**). The signals in the EPR spectra are different and depend on the percent of admixture of oxo–titanium(IV) complex and time of measurements. This is a significant difference from previously studied systems, where there was only a difference in intensities, which seems obvious [1]. Spectra registered for composites of PCL with 10, 15 and 20% of (**1**) are presented in Figure 5. The EPR parameters and types of observed paramagnetic species (ROS) are summarized in Table 2.

## 4. Discussion

In the present study, in vitro biocompatibility of PCL and PCL + (**1**) composite was evaluated using two cell lines: murine fibroblast cell line L929 and HUVEC cells, which represent cells essential for the proper functioning of materials with antithrombotic properties. The biocompatibility of PCL and PCL + (**1**) foils was estimated according to the ISO 10993-5 and ISO 10993-12 standards using two different methods: extract test and direct contact to the foils. The extract assay estimated the cytotoxicity of any leachable products from the materials after 72 h-lasting of incubation in culture media. Although ISO 10993-12 standard allows for extraction of materials in culture medium at 37 °C only for 24 h, we decided in our experiments to extend the extraction time to 72 h, since the tested samples may have long-term contact with the biological microenvironment after implantation. According to the ISO norm, tested samples that decrease cell viability below 70% are considered as toxic. The results from the MTT assay showed that the extracts derived from PCL samples did not decrease the viability of L929 and HUVEC cells in all tested time variants of incubation. In contrast, the cell viability was significantly decreased following the PCL- and PCL + (**1**)-derived extraction media challenge in time-dependent and concentration-dependent manners. Generally, among the tested extract, the statistically significant cytotoxic effect on L929 fibroblasts was only observed for the cells incubated with the 100% extract derived from PCL + (**1**) 15 wt.% samples during 72 h of incubation and from PCL + (**1**) 20 wt.% specimens after 48 h and 72 h of stimulation. HUVEC cells were more sensitive to the toxic effect of extracts, since their viability was reduced below 70% in the cases of the incubation with 50% extracts from PCL + (**1**) 15 wt.% and PCL + (**1**) 20 wt.% foils for 72 h. This observation suggests that the toxic effect was reduced with dilution of the extracts and lower concentration of TOC. In addition, the most concentrated level of tested extract media (undiluted extracts) maximally reduced viability of L929 fibroblasts and HUVEC cells up to 55% and 44%, respectively, which is considered as a moderate cytotoxic response according to the ISO 10993-5 standard (range 30%–59%) [11,12]. 

Although the cytotoxic effect of the undiluted extracts from PCL and PCL + (**1**) samples (containing 15% and 20% TOC) was moderate, in a clinical condition it may be even lower, as the extracts would be diluted by the surrounding tissue fluid [13,14]. Partially, this assumption can be explained by the results of the viability of cells incubated with 100% extracts, which were presented as the changes in absorbance values measured over time. These findings showed that L929 cells incubated with PLC-/PCL + (**1**)-derived undiluted extract, similar to the cells treated with the PCL-derived extract, proliferated over time, in what was observed as an increase in absorbance level. In contrast, the increase in proliferation level of HUVEC cells over time was observed only for the PCL-derived undiluted extracts, which confirms that endothelial cells are more sensitive to the toxic effect of the extracts derived from PCL/PCL + (**1**) specimens. 

Additional confirmation of such conclusions are the results of the cell viability assessment during the direct cytotoxicity assay. L929 fibroblasts cultured on the surface of PCL/PCL + (**1**) foils proliferated on all tested specimens over time in the PCL samples. In contrast, the surfaces of all tested materials did not induce the proliferation of HUVEC cells. Importantly, this effect was also observed for endothelial cells cultivated on the PCL specimens, which, in the extract assays, have not shown potential cytotoxicity. The main potential explanation of this phenomenon was the lack of use of the Attachment Factor Protein for coating of foil surfaces before seeding cells, which promotes attachment and growth of many different lines of endothelial cells, including HUVEC cells. Relou et al. have demonstrated that HUVEC grown on uncoated surfaces, even on plastic, showed low spontaneous proliferation [15]. Moreover, there is evidence that adhesion, spreading, viability and proliferation rates of vascular endothelial cells are remarkably enhanced on the scaffolds coated with fibronectin, collagen, gelatine (a molecular derivative of collagen) or laminin compared to uncoated surface [16,17,18,19]. Additionally, bare PCL fibers are too hydrophobic to efficiently induce endothelial cell adherence and proliferation [20]. These results indicate that the PCL specimens coated with different proteins should be considered as a more biocompatible scaffold to support the growth of endothelial cells than uncoated PCL samples. In our experiments, we decided not to use Attachment Factor Proteins in order not to modify the surface of the tested samples and to ensure the conditions of the experiments were as close to physiological as possible.

Biocompatibility of the tested PCL/PCL + (**1**) was also evaluated with the analysis of SEM micrographs, which presented the morphology and proliferation level of the L929 fibroblasts and HUVEC cells. These data confirmed the result of cell viability during assessment of direct cytotoxicity and showed that L929 fibroblasts effectively attached to the surface of PCL + (**1**) 10 wt.% and produced the extracellular matrix. Moreover, L929 cells had an elongated shape, preparing cells for division, and the number of the growing cells increased over time to such levels that, after 72 h, fibroblasts started to grow in layers on top of each other. L929 fibroblasts also created numerous filopodia between themselves and the filopodia that attached the cells to the foil’s surface. These thin actin-rich plasma membrane protrusions play a fundamental role in cell attachment, migration, proliferation and cell–cell interactions [21,22] In contrast, SEM micrographs of HUVEC cells demonstrated low numbers of endothelial cells attached to the surface of PCL+ (**1**) 10 wt.%. Moreover, these cells had rounded shapes and their number did not change over time.

The results from the cell viability assays and SEM analysis clearly indicate the significantly higher toxic effect of the PCL/PCL + (**1**) samples on HUVEC cells than L929 fibroblasts. We presume that this phenomenon is associated with the presence of acetylsalicylic acid ligands and their potential release into the microenvironment during cell culture. The numerous reports showed that a high concentration of aspirin (in range of 1–6 mM) significantly decreases viability, inhibits proliferation rate and causes apoptosis of HUVEC cells and other endothelial cell lines [23,24,25]. However, therapeutic aspirin concentrations (0.5 mM) have no detectable effect on endothelial cell viability or proliferation [25]. In contrast, Nordin et al. demonstrated that L929 fibroblasts exposed to acetylsalicylic acid even for 13 days continued to proliferate during the entire period, although at a reduced rate [26]. 

The potential explanation of cytotoxic effect of the PCL/PCL + (**1**) foils can be associated with the results obtained from EPR spectroscopy, which revealed the presence of ROS on the specimens’ surfaces. The richest EPR spectrum was registered for PCL + (**1**) 20 wt.% samples and, at the same time, these samples induced the highest cytotoxicity. It is well established that ROS serve as cell signaling molecules for normal biologic processes. However, the generation of ROS can also provoke damage to multiple cellular organelles and processes, which can ultimately disrupt normal physiology and lead to cell death [27]. Moreover, several studies have shown that ASA increases the production of ROS, leading to the decrease in cell viability. Raza et al. demonstrated that ASA induced oxidative stress that affected G0/G1 cell cycle arrest, mitochondrial dysfunction and apoptosis in human hepatoma HepG2 cancer cells [28]. These same authors also showed that the addition of ASA increased oxidative stress in the J774.2 mouse macrophages treated with liposaccharide (LPS) in comparison with the cells stimulated only with LPS, in which markedly-induced ROS production was also observed. This effect was accompanied by the inhibition of mitochondrial respiratory complexes and ATP production, and a higher apoptotic cell death level [29]. The ASA-induced ROS production also increased permeability of intestinal epithelial cells, leading to their damage [30]. On the other hand, there is also evidences that ASA is able to protect the cells from oxidative stress, which was observed in endothelial cells [31,32] or cerebral cortical astrocytes [33]. All these results suggest that nontoxic doses of ASA might protect the cells from injury induced by ROS, while, at the same time, high concentrations of ASA can induce ROS-dependent cell death. Although additional experiments are required, we concluded that, for the preparation of PCL/PCL + (**1**) foils, TOCs should be used at concentrations that do not cause significant severe cytotoxic effects, at least partially associated with ROS generation.

Considering the relatively well-known mechanism of the photocatalytic action of TiO_2_-based materials, it can be assumed that, after visible light photoexcitation of studied samples, which was proved in our earlier studies, the ROS can be generated on their surface [34]. Therefore, the microbiocidal activity estimation of the produced composites (containing 20 wt.% TOC) were preceded by an analysis of their EPR spectra, which allowed for the detection of ROS formation and their identification. Typical EPR spectra of PCL/PCL + (**1**) samples are shown in Figure 5. The richest EPR spectrum was registered for PCL + (**1**) 20 wt.% TOC (Figure 5C). The characteristic anisotropic signals for O^−^ radicals were found (Figure 5, Table 2). The formation of O^−^ type paramagnetic centers consists of removing the electron and stabilizing the radical formed by reduction of titanium(IV) to titanium(III) [35]. Due to a positive spin-orbit coupling constant, titanium(III) shows EPR signals at lower than radical g-values, ca. 1.99 and below [34,35,36,37,38]. Assuming the expected anisotropy of the titanium(III) signals, one can expect lines with even lower g-factors. Unfortunately, the respective signals are not visible, probably due to their much greater width compared to the other signals and, consequently, even lower intensity. According to the literature data, the maximum g-factor for O_2_^−^ is greater than for O^−^ species and is expected at ca. 2.025 [36]. In our case, such a signal was not detected. Over time, after about a month, the spectrum for PCL + (**1**) 20 wt.% drastically changes and becomes similar to the spectrum for PCL + (**1**) 10 wt.% (Figure 5A). The radical and titanium signal intensities decrease significantly. The spectrum of PCL + (**1**) 10 wt.% (Figure 5A) is dominated by the signal of an unknown radical. Interestingly, the same signal alone occurs in the spectra of the substrate and the sample of PCL + (**1**) 15 wt.% after a month (Figure 5B). Fresh sample of PCL + (**1**) 15 wt.% shows EPR spectrum, which is similar to that of PCL + (**1**) 20 wt%. 

As shown previously by UV–Vis-DRS measurements, sterilization of the PCL and PCL + TOC systems using UVC light before the antimicrobial assay did not activate the tested samples [3]. Further irradiation of PCL enriched with TOC grains using natural indoor light directly before inoculation with microorganisms led to ROS generation on their surfaces. Based on the results discussed above, we can assume that the ROS generated on the surface of the polymer enriched with TOC grains, under exposition to the visible light, is the main antimicrobial mechanism of action. As mentioned earlier, the mechanisms for the biocidal action of TiO_2_-based photocatalysts against bacterial strains are associated with the generation of ROS, including superoxide radical anion (O_2_^−^·), hydrogen peroxide (H_2_O_2_) and hydroxyl radicals (OH·), which cause oxidative damage to living cells [39,40]. All the studied PCL + TOC foils strongly (>99%) inhibited the growth of tested Gram-positive bacteria *S. aureus*, and those with higher contents of TOC grains also of Gram-negative bacteria of *E. coli*. Overall, lower activity against *E. coli*, observed for the PCL + (**1**) 10 wt.%, could be related to its worst photocatalytic properties. The photocatalytic activity of the PCL + (**1**) 15 and 20 wt.% foils was higher compared to 10% wt.%, therefore, stronger inhibition of microbial growth was observed on their surfaces. Although generation of ROS is the main proposed mechanism of antimicrobial activity of TiO_2_-based nanomaterials and Ti(IV) complexes, the other mechanism of action should be also considered. Strong inhibitory effects of different metal-based nanomaterials towards a broad spectrum of bacterial strains are a well-known mechanism. It is claimed that the biocidal effect of such materials, including titanium oxide nanoparticles (TiONPs), results from the “electromagnetic” attraction between the negatively charged microbial cell and positive charged metal oxide nanoparticles [41]. It was reported that the density of the negative charge of the lipopolysaccharide-coated outer surface of Gram-negative *Escherichia coli* was seven times larger than that found in the case of the protein surface layer of Gram-positive *Lactobacillus* [42]. Although the *C. albicans* cell wall is also negatively charged, it has a different structure and composition than prokaryotic ones; it is much thicker and composed from several layers made mainly of β-glucans and chitin [43,44]. while in Gram-positive bacteria the main composite of the call wall is peptidoglycan polysaccharide and in Gram-negative bacteria outer phospholipid membrane [42].

From an antimicrobial, anticandidal activity, cytotoxicity and photocatalytic activity point of view, freshly obtained samples are crucial. Therefore, according to the analysis of EPR spectra, the presence of O^−^ and unknown radicals are responsible for their above-mentioned properties. Interestingly, the unknown radical is transferred to the PCL + TOC composites from the substrate.

## 5. Conclusions

It was necessary to assess the possible cytotoxicity of the tested composites of PCL + (1), which is extremely important in the case of potential biomedical applications of these systems. The toxic effect was reduced with dilution of the extracts and lower concentrations of TOC. In addition, the most concentrated level of tested extract media (undiluted extracts) maximally reduced viability of L929 fibroblasts and HUVEC cells up to 55% and 44%, respectively, which is considered as a moderate cytotoxic response according to the ISO 10993-5 standard (range 30%–59%) [26,27]. Analysis of SEM micrographs, which presented the morphology and proliferation levels of the L929 fibroblasts and HUVEC cells, confirmed the result of cell viability during assessment of direct cytotoxicity and showed that L929 fibroblasts effectively attached to the surface of PCL + (**1**) 10 wt.% and produced the extracellular matrix. The results from the cell viability assays and SEM analysis clearly indicate the significantly higher toxic effect of the PCL/PCL + (**1**) samples on HUVEC cells than L929 fibroblasts. We presume that this phenomenon is associated with the presence of acetylsalicylic acid ligands and their potential release into the microenvironment during cell culture. The potential explanation of cytotoxic effect of the PCL/PCL + (**1**) foils can be associated with the results obtained from EPR spectroscopy, which revealed the presence of ROS on the specimens’ surfaces. Although additional experiments are required, we concluded that, for the preparation of PCL/PCL + (**1**) foils, TOCs should be used at concentrations that do not cause significant severe cytotoxic effects, at least partially associated with ROS generation.

Analysis of EPR spectra of the above-mentioned samples proved that photoexcitation of their surface led to the formation of different paramagnetic species, mainly O^−^, which slowly disappears over time. The destructive effect of radicals on bacteria and cells has been proven. From the studied composite samples, the strong biocidal activity (against *E. coli* and *S. aureus* strains) and weak inhibition of yeast (*C. albicans*) growth was found for analyzed composites, especially those with 15 and 20 wt.% of TOC content. 

## Figures and Tables

**Table 1:** Reduction index of microbial growth.

	Microorganisms				
Sample	*Escherichia coli* ATCC 8739	*Escherichia coli* ATCC 25922	*Staphylococcus aureus* ATCC 6538	*Staphylococcus aureus* ATCC 25923	*Candida albicans* ATCC 10231
	R	R	R	R	R
PCL	+0.15 *	0.2	+0.3 *	+0.4 *	+1.0 *
PCL + (1) 10 wt.%	1.0	1.8	4.5	4.6	+0.1 *
PCL + (1) 15 wt.%	2.1	2.8	4.5	4.6	1.1
PCL + (1) 20 wt.%	2.0	3.7	4.5	4.6	0.6

Reduction factor (R) ≥ 2 indicates biocidal activity of a specimen (at least 99% reduction of microbial growth). * Increase in the number of microorganisms in the presence of PCL.

**Table 2:** EPR data for pure TOC (**1**) and PCL with 10 (a), 15 (b) and 20% (c) TOC.

Sample	g-Factors	Species
(1)	2.0036	Radical
PCL + (1) 10 wt.%	2.017; 2.0036; 1.993	O^−^ + Radical + Ti(III)
PCL + (1) 15 wt.%	2.0036	Radical
PCL + (1) 20 wt.%	2.017; 2.009; 2.002; 1.992	O^−^ + Radical + Ti(III)

The samples were exposed to visible light prior to measurement.

## Data Availability

Data are contained within the article.
